# The *CYP4G15*-*SORD*-like-associated pathway promotes ovarian ZIKV infection and egg-associated viral RNA detection in *Aedes albopictus*

**DOI:** 10.3389/fcimb.2026.1910501

**Published:** 2026-08-27

**Authors:** Mei Chen, Yajing Zhang, Wenwei Zhong, Mei Hong, Jinbao Gu, Zetian Lai

**Affiliations:** 1 Center of Laboratory Medicine, Shenzhen Nanshan People’s Hospital (Affiliated Sixth Hospital of Shenzhen University), Shenzhen, Guangdong, China; 2 School of Public Health, Southern Medical University, Guangzhou, Guangdong, China; 3 Medical Laboratory, The Third Affiliated Hospital of Shenzhen University, Shenzhen, Guangdong, China; 4 College of Laboratory Medicine, Dalian Medical University, Dalian, Liaoning, China; 5 Guangdong Provincial Key Laboratory of Infection Immunity & Inflammation, Department of Pathogen Biology, Shenzhen University Medical School, Shenzhen, Guangdong, China

**Keywords:** *Aedes albopictus*, *CYP4G15*, metabolic regulation, ovarian infection, *SORD-like*, vertical transmission, Zika virus

## Abstract

Zika virus (ZIKV) can persist in mosquito populations through both horizontal and vertical transmission, and successful ovarian infection is a prerequisite for transgenerational spread. However, the mosquito host factors that regulate ovarian permissiveness and vertical transmission remain poorly characterized. Here, we investigated the role of cytochrome P450 4G15 (*CYP4G15*) in ZIKV infection and vertical transmission in *Aedes albopictus* and identified an associated downstream pathway involving *SORD-like*, a predicted sorbitol dehydrogenase-like gene. Expression profiling revealed that *CYP4G15* exhibits distinct tissue-, developmental-, blood-feeding-, and infection-responsive patterns, with transient induction in ovaries during the early phase of ZIKV infection. RNA interference-mediated silencing of *CYP4G15*, followed by transcriptomic analysis and RT-qPCR validation, identified *SORD-like* (LOC109415972) as a candidate *CYP4G15*-associated gene. *SORD-like* was highly expressed in the fat body, strongly induced after blood feeding, and responsive to ZIKV challenge in ovarian tissues. Functional assays showed that knockdown of *SORD-like* significantly reduced early ovarian infection rate and viral RNA accumulation, while also decreasing conditional progeny egg-pool positivity rate and minimum egg infection rate, indicating reduced ZIKV RNA detection in progeny egg pools. *SORD-like* silencing did not alter *CYP4G15* transcript levels, whereas *CYP4G15* overexpression was associated with increased *SORD-like* transcript abundance and functionally compensated for the ovarian viral RNA phenotype caused by *SORD-like* knockdown. Together, these findings support a functional association between *CYP4G15, SORD-like*, and ovarian ZIKV infection, but do not establish direct transcriptional regulation or a strict linear epistatic relationship. Our findings reveal a *CYP4G15*–*SORD-lik*e-associated metabolic-regulatory pathway that links mosquito physiology to arbovirus persistence. Given that *CYP4G*-related pathways are often associated with environmental adaptation and insecticide resistance-associated physiology, this pathway may represent an important point of intersection between vector fitness and viral transmission. It may therefore serve as a candidate target for reducing egg-associated ZIKV RNA detection and ZIKV maintenance in *Aedes albopictus* populations.

## 1 Introduction

Zika virus (ZIKV) is a mosquito-borne flavivirus of major public health importance. In addition to causing self-limiting febrile illness, ZIKV infection is associated with severe neurological complications, including Guillain–Barré syndrome in adults and congenital abnormalities such as microcephaly in infants (Duffy et al., 2009; Krauer et al., 2017). With no widely implemented antiviral therapies currently available, prevention still relies heavily on vector surveillance and mosquito control (De Jong and Grobusch, 2025). Elucidating mosquito host factors that support viral persistence, particularly through vertical transmission, is therefore critical for understanding ZIKV transmission dynamics and for developing new intervention strategies.

*Aedes albopictus*, one of the most invasive mosquito species worldwide, is a competent vector for ZIKV, dengue virus, and chikungunya virus (Paupy et al., 2009; Bonizzoni et al., 2013). In addition to horizontal transmission via blood feeding, arboviruses can also persist in mosquito populations through vertical transmission, in which infected females pass the virus to their offspring (Janjoter et al., 2024). This route is epidemiologically important because it may enable viral maintenance during inter-epidemic periods or under unfavorable environmental conditions when mosquito–host contact is limited (Janjoter et al., 2024). Vertical transmission of flaviviruses is constrained by sequential barriers involving ovarian infection, follicular or oocyte entry, and persistence through embryonic development (Anderson and Spielman, 1971; Nag et al., 2021; Lai et al., 2020). Blood-meal-induced ovarian changes and longer extrinsic incubation can increase ovarian or filial infection, whereas the effect of gonotrophic cycle is context dependent (Nag et al., 2021; Manuel et al., 2020; Sánchez-Vargas et al., 2018). In *Aedes albopictus*, reported overall or egg-level ZIKV vertical infection estimates are generally low (approximately 0–2.1%) and vary with mosquito population, viral dose, gonotrophic cycle, and the metric used (Lai et al., 2020; Zimler and Alto, 2023). For ZIKV, successful ovarian infection is a prerequisite for transgenerational spread, as the virus must invade reproductive tissues before accessing developing oocytes (Janjoter et al., 2024; Lai et al., 2020). However, the host molecular determinants that regulate ovarian permissiveness and vertical transmission efficiency in *Ae. albopictus* remain poorly understood (Janjoter et al., 2024; Lai et al., 2020).

Cytochrome P450 enzymes constitute a large family of heme-containing monooxygenases involved in the metabolism of endogenous compounds and xenobiotics in insects (Qiu et al., 2012; Feyereisen, 2020). The *CYP4G* subfamily is distinctive in that it is primarily associated with cuticular hydrocarbon biosynthesis, desiccation resistance, and chemical communication rather than classical detoxification (Qiu et al., 2012; Feyereisen, 2020). In *Aedes* mosquitoes, P450-associated pathways also play important roles in insecticide resistance and adaptive physiology (Lien et al., 2019; Zulfa et al., 2022), and emerging evidence suggests that these physiological programs may influence arbovirus transmission traits in a context-dependent manner (Huang et al., 2019; Merkling et al., 2025). Whether specific *CYP4G* family members contribute to ovarian ZIKV infection and vertical transmission, however, has not been explored.

Previous studies from our laboratory have shown that *CYP4G15* expression is induced following ZIKV infection in *Ae. albopictus*, and that silencing this gene reduces viral RNA burden in ovaries and impairs infection-related parameters in progeny eggs (Lai et al., 2020). Transcriptomic profiling after *CYP4G15* knockdown further identified LOC109415972, hereafter referred to as *SORD-like*, as a reproducibly downregulated candidate. Among the three candidates validated by both RNA-seq and RT-qPCR, 109415972 had the highest FPKM value and encodes a sorbitol dehydrogenase-like protein (*SORD-like*) involved in energy metabolism and redox balance, whereas 109432313 has unknown function and 109429642 encodes a retinol-binding protein with a less clear link to viral replication. Thus, 109415972 was selected for further study. Given the close link between metabolic remodeling, mosquito reproduction, and arbovirus infection, and because *SORD-like* is dynamically regulated across blood-feeding and developmental contexts, this gene emerged as a plausible downstream effector.

In the present study, we investigated the *CYP4G15*–*SORD-like*-associated pathway in ovarian ZIKV infection and vertical transmission in *Aedes albopictus*. By integrating expression profiling, RNA interference, transcriptome-based candidate screening, functional infection assays, vertical transmission experiments, and regulatory rescue-like analyses, we asked whether *SORD-like* functions downstream of *CYP4G15* and whether this pathway influences the timing and efficiency of ovarian infection. More broadly, this study addresses whether a host pathway associated with adaptive mosquito physiology also contributes to arbovirus maintenance across generations.

## 2 Materials and methods

### 2.1 Mosquitoes and rearing conditions

A laboratory colony of *Aedes albopictus* was used throughout this study. The colony was originally established from mosquitoes collected in Foshan, Guangdong Province, China, and was subsequently maintained under standard insectary conditions after being provided by the Guangzhou Center for Disease Control and Prevention. Mosquitoes were reared at 27 ± 1 °C, 70–80% relative humidity, and a 16 h:8 h light: dark photoperiod. Eggs were deposited on moist filter paper, air-dried, and stored for colony maintenance. Larvae were reared in dechlorinated water and fed commercial turtle food. Pupae were transferred to small cups containing dechlorinated water and allowed to emerge in screened adult cages. Adult mosquitoes were provided with 10% glucose solution ad libitum.

Unless otherwise specified, 3–5-day-old adult females were used for gene expression profiling, RNA interference, viral infection, and rescue experiments. For vertical transmission assays, newly emerged females were allowed to mate with males for 4–5 days prior to treatment. Blood feeding was performed using a Hemotek membrane feeding system with defibrinated sheep blood.

### 2.2 Cells, virus, and biosafety

The *Aedes albopictus* C6/36 cell line was used for amplification of ZIKV stocks. Cells were maintained in RPMI 1640 medium supplemented with 10% fetal bovine serum and 1% penicillin–streptomycin at 28 °C. The ZIKV strain used in this study was PRVABC59 (GenBank accession no. KU501215), kindly provided by Tsinghua University.

For virus stock preparation, C6/36 cells grown to approximately 80% confluence were washed with phosphate-buffered saline (PBS) and inoculated with virus-containing supernatant in medium supplemented with 2% fetal bovine serum. After 2 h of adsorption, fresh maintenance medium was added, and infected cultures were incubated for 7–10 days. The viral RNA copy number in the harvested supernatant was determined by absolute quantitative RT-qPCR using a standard curve generated from a plasmid containing the ZIKV NS1 gene. Virus stocks with a concentration of >1×10^5^ copies/μL were used for subsequent experiments. The ZIKV stock was quantified by absolute RT-qPCR using an NS1 plasmid standard curve. Infectious titers determined by plaque, focus-forming, or TCID_50_ assays were not available; therefore, the reported viral doses represent RNA copy numbers rather than infectious units. All procedures involving infectious ZIKV were performed in a certified biosafety cabinet in accordance with institutional biosafety requirements.

### 2.3 RNA extraction, reverse transcription, and quantitative PCR

Total RNA was extracted from whole mosquitoes, dissected tissues, cultured cells, ovarian samples, or egg pools using AG RNAex Pro Reagent according to the manufacturer’s instructions. Briefly, samples were homogenized in TRIzol-type reagent, followed by phase separation with chloroform, RNA precipitation with isopropanol, washing with 75% ethanol, and resuspension in RNase-free water. RNA concentration and purity were assessed using a NanoDrop spectrophotometer. RNA samples with A260/A280 ratios between 1.8 and 2.1 were used for subsequent analyses.

For cDNA synthesis, 1 μg of total RNA was reverse-transcribed using the TransScript One-Step gDNA Removal and cDNA Synthesis SuperMix kit in a 20 μL reaction volume. Quantitative PCR (qPCR) was performed on an Applied Biosystems QuantStudio 6 Real-Time PCR System using PerfectStart Green qPCR SuperMix. Ribosomal protein S7 (*Aalrps7*) was used as the internal reference gene for normalization of mosquito transcripts. Relative gene expression was calculated using the 2^−ΔΔCt method (Livak and Schmittgen, 2001). The specificity of each primer pair was validated by melt curve analysis, which showed a single peak for all primers, confirming the absence of primer-dimers or non-specific amplification.

For qualitative detection of ZIKV, endpoint RT-PCR was first performed using ZIKV-specific primers. Samples yielding the expected amplicon were considered ZIKV-positive and were subsequently subjected to qPCR for viral RNA copy-number determination using a plasmid-derived standard curve. For absolute quantification, a standard curve was generated using a linearized plasmid containing the ZIKV NS1 gene fragment (296 bp) inserted into the pLB vector (total plasmid length: 3, 270 bp). The qPCR amplicon for viral quantification corresponds to a 141 bp region within the NS1 gene. Plasmid copy numbers were calculated based on the total plasmid length (3, 270 bp), and a standard curve ranging from 6.79×10^5^ to 6.79×10^9^ copies/μL was used for absolute quantification of viral RNA in experimental samples. Primer sequences used in this study are provided in **Supplementary Table 1**.

### 2.4 Expression profiling of *CYP4G15* and *SORD-like*

To characterize the expression patterns of *CYP4G15* and *SORD-like* (LOC109415972), tissue-specific, blood-feeding-associated, developmental, and infection-responsive profiles were analyzed by RT-qPCR.

For tissue distribution analysis, heads, thoraces, fat bodies, midguts, ovaries, and salivary glands were dissected from non-blood-fed, unmated adult females at 3–4 days post-emergence in ice-cold PBS. Twenty tissues of the same type were pooled for each biological replicate.

For blood-feeding time-course analysis, whole females were collected before blood feeding and at 12, 24, 36, 48, and 72 h post-blood meal. Ten mosquitoes were pooled per biological replicate.

For developmental profiling, eggs at 0–2, 2–4, 4–8, 8–12, 12–24, and 24–48 h after oviposition, first- to second-instar larvae, third- to fourth-instar larvae, female pupae, male pupae, adult females, and adult males were collected. Each biological replicate consisted of 30–50 eggs, 12 larvae, 12 pupae, or 10 adults, depending on developmental stage.

For infection-responsive expression analysis of *CYP4G15* and *SORD-like*, adult females were intrathoracically injected with 500 nL of ZIKV suspension. Ovaries were collected at 2, 4, and 7 days post-infection, and 30 ovaries were pooled for each biological replicate. All expression profiling experiments were performed with three independent biological replicates.

### 2.5 Synthesis of double-stranded RNA

Gene-specific double-stranded RNA (dsRNA) was synthesized for *CYP4G15* and *SORD-like*. Target fragments were amplified from *Ae. albopictus* cDNA using gene-specific primers containing T7 promoter sequences at the 5′ ends. The target regions corresponded to a 655-bp fragment of *CYP4G15*, a 417-bp fragment of *SORD-like*, and a 500-bp fragment of GFP, which served as the negative control.

PCR products were purified, sequence-verified, and used as templates for *in vitro* transcription with the T7 RiboMAX Express RNAi System according to the manufacturer’s protocol. After transcription, complementary RNA strands were annealed, and residual single-stranded RNA and template DNA were removed by RNase and DNase treatment. dsRNA was precipitated with sodium acetate and isopropanol, washed with 70% ethanol, dissolved in RNase-free water, quantified spectrophotometrically, and examined by agarose gel electrophoresis. Aliquots were stored at −80 °C until use.

To minimize potential off-target effects, dsRNA target sequences were subjected to BLASTn analysis against the *Ae. albopictus* genome, and no significant homology to unrelated genes was detected.

### 2.6 RNA interference in adult mosquitoes

For *CYP4G15* knockdown, dsRNA was diluted to 2, 000 ng/μL and intrathoracically injected into cold-anesthetized 3–5-day-old adult females using pulled glass capillary needles. Each mosquito received 500 nL of dsRNA. Three groups were included: ds*CYP4G15*-injected mosquitoes, dsGFP-injected mosquitoes as the negative control, and uninjected controls. After injection, mosquitoes were maintained on 10% glucose solution. Knockdown efficiency was assessed by RT-qPCR at 1, 2, 3, 4, and 5 days post-injection. Eight mosquitoes were pooled for each biological replicate. Day 3 post-injection was selected for transcriptome sequencing because silencing efficiency was robust and inter-replicate variability was relatively low at this time point.

For *SORD-like* knockdown in downstream functional experiments, dsRNA was synthesized and delivered in the same manner, either alone or in combination with ZIKV, depending on the experimental design. Knockdown efficiency was verified by RT-qPCR at 2, 4, and 7 days post-injection in parallel experiments conducted without ZIKV infection, using the same dsRNA concentration, injection route, and injection volume. These time points were chosen to cover the early, middle, and late phases of the RNAi effect, as silencing typically lasts for approximately 7 days in mosquitoes. For ovarian functional assays, knockdown efficiency was confirmed in dissected ovarian tissues from parallel groups to ensure that gene silencing occurred in the target organ. Knockdown was not independently re-validated in every ZIKV-infected functional-assay cohort. For transcriptomic screening and initial validation, RNA was extracted from whole mosquitoes. Fat body knockdown efficiency was not independently assessed, as ovarian tissue was the primary focus of this study.

### 2.7 Transcriptome sequencing and candidate-gene screening

To identify genes potentially regulated by *CYP4G15*, transcriptome sequencing was performed following RNAi-mediated silencing of *CYP4G15*. Based on the knockdown time-course, day 3 post-injection was selected because silencing efficiency was robust and inter-replicate variability was relatively low at this time point. Total RNA from ds*CYP4G15*-injected mosquitoes, dsGFP-injected mosquitoes as the negative control, and uninjected controls was extracted and submitted to BGI (Shenzhen, China) for mRNA sequencing on the DNBSEQ platform with paired-end 150-bp reads. Each group contained three biological replicates.

Raw reads were filtered to remove adapter contamination, reads containing excessive unknown bases, and low-quality reads. Clean reads were mapped to the *Aedes albopictus* reference genome (GCF_006496715.1_Aalbo_primary.1.v2201) using HISAT2, and transcript abundance was quantified using RSEM. Differentially expressed genes were identified using DESeq2 and screened using the following criteria: |log2(fold change)| > 2, q < 0.05, and FPKM > 10. Genes showing excessive within-group variation or strong sequence homology to *CYP4G15* were excluded from downstream analysis. Candidate genes were further validated by RT-qPCR. LOC109415972 (*SORD-like*) was selected for subsequent functional analysis based on consistency between RNA-seq and RT-qPCR results, functional annotation, and biological relevance to the ZIKV infection model. The complete differential-expression table (gene ID, log2 fold change, P value, adjusted P value, and FPKM values) is provided in **Supplementary Table 3**.

### 2.8 Construction of the ZIKV standard plasmid

To quantify viral RNA levels, a plasmid-based ZIKV standard was generated. Viral RNA was extracted from ZIKV-infected C6/36 cells and reverse-transcribed into cDNA. A ZIKV-specific fragment was amplified by PCR, purified, and ligated into the pLB cloning vector. Recombinant plasmids were transformed into TOP10 competent *Escherichia coli* cells. Positive clones were identified by colony PCR and confirmed by Sanger sequencing. Plasmids were purified, linearized by XhoI digestion, and quantified spectrophotometrically.

Plasmid copy number was calculated based on DNA concentration and fragment length. Serial dilutions were prepared to generate a qPCR standard curve, and viral RNA copy numbers in experimental samples were determined according to Ct values relative to this standard curve.

### 2.9 Functional analysis of *SORD-like* during ovarian infection

To determine whether *SORD-like* contributes to ovarian ZIKV infection, adult females were intrathoracically co-injected with dsRNA and virus. Three groups were included: ds*SORD-like* + ZIKV, dsGFP + ZIKV as the negative control, and ZIKV-only controls. For these assays, ds*SORD-like* was diluted to 4, 000 ng/μL and mixed 1:1 with freshly prepared ZIKV suspension immediately before use. Each mosquito received a total inoculum of 500 nL. To ensure equal virus doses across all groups, the ZIKV-only control group received 500 nL of a 1:1 mixture of virus suspension and RNase-free water, whereas the dsRNA–ZIKV co-injection groups (dsGFP + ZIKV and ds*SORD-like* + ZIKV) received 500 nL of a 1:1 mixture of virus suspension and the respective dsRNA solution (250 nL each). This ensured that all groups received the same volume of virus suspension and total inoculum.

At 4, 7, and 10 days post-infection, ovaries were dissected and examined for ZIKV infection. Ovarian infection was first assessed by endpoint RT-PCR, and the proportion of ZIKV-positive ovaries was used to calculate the ovarian infection rate. Positive ovarian samples were subsequently analyzed by qPCR to determine viral RNA copy number. Three independent biological replicates were performed for each time point. For each time point and treatment group, 30 individual ovaries were examined (10 ovaries per biological replicate, N = 3).

Because these assays were based on intrathoracic inoculation, the resulting phenotype primarily reflects systemic and ovarian stages of infection after bypassing the natural midgut infection barrier. Therefore, the findings should be interpreted as reflecting post-midgut events, and the relevance of this pathway to natural oral infection remains to be determined.

### 2.10 Assessment of egg-associated ZIKV RNA detection

To investigate the effect of *SORD-like* on ZIKV RNA detection in progeny eggs, newly emerged females were allowed to mate with males for 5 days and were then intrathoracically co-injected with ds*SORD-like* + ZIKV, dsGFP + ZIKV, or ZIKV alone. One day after injection, females were blood-fed using a Hemotek membrane feeder. Fully engorged females were isolated individually, maintained on 10% glucose solution, and provided with oviposition cups lined with moist filter paper beginning approximately 3 days after injection.

After oviposition, ovaries from each female were collected and screened for ZIKV. Only egg batches derived from females with ZIKV-positive ovaries were included in downstream analyses. Before RNA extraction, eggs were surface-decontaminated with sodium hypochlorite solution and washed 3 times with sterile RNase-free water to minimize externally associated maternal viral RNA contamination. Egg pools were screened by endpoint RT-PCR, and positive pools were further quantified by qPCR when required.

Outcomes were evaluated at the maternal ovary and egg-pool levels. Ovarian positivity rate was defined as the number of ZIKV-positive maternal ovaries divided by the total number of ovaries examined. Conditional egg-pool positivity was defined as the number of ZIKV RNA-positive egg pools divided by the total number of egg pools tested among egg batches derived from females with ZIKV-positive ovaries. The minimum egg infection rate was calculated as the number of ZIKV RNA-positive egg pools divided by the total number of eggs represented in the tested pools. The total number of eggs examined in each group was also recorded (Lai et al., 2020). Because eggs were tested in pools and only egg batches from ovarian-positive females were included, these outcomes do not represent an overall vertical-transmission rate or the number of individually infected eggs.

### 2.11 Construction of overexpression plasmids

The full-length coding sequences of *CYP4G15* and *SORD-like* were separately amplified from *Ae. Albopictus* cDNA using high-fidelity polymerase with gene-specific primers. The mosquito expression vector AePUB-RFP, kindly provided by Prof. Jun-Hong Chen, was linearized with NotI and purified from agarose gel. The purified *CYP4G15* and *SORD-like* inserts were individually assembled into the linearized vector using a seamless cloning strategy to generate AePUB-*CYP4G15* and AePUB-*SORD-like*, respectively.

Recombinant clones were screened by colony PCR, confirmed by Sanger sequencing, and expanded for plasmid preparation. Endotoxin-free plasmid DNA was purified and used for intrathoracic injection into mosquitoes. For overexpression efficiency validation, RNA was extracted from dissected ovarian tissues (30 ovaries per biological replicate, N = 3) at 2 and 4 days post-injection, and RT-qPCR was performed using gene-specific primers. *SORD-like* overexpression efficiency is shown in **Supplementary Figure 1**. Mosquitoes injected with the empty PUB vector and uninjected controls served as controls.

### 2.12 Analysis of the functional association between *CYP4G15* and *SORD-like*

To examine the directional functional association between *CYP4G15* and *SORD-like*, two complementary experiments were performed.

First, *SORD-like* was knocked down in adult females, and ovaries were collected at 2, 4, and 7 days post-injection. For each time point, RNA was extracted from pools of 30 ovaries per biological replicate (N = 3). RT-qPCR was used to confirm *SORD-like* silencing and to examine whether reduced *SORD-like* expression affected *CYP4G15* transcript levels.

Second, to assess functional compensation, mosquitoes were injected with the *CYP4G15* or *SORD-like* overexpression plasmids, alone or combined with the corresponding dsRNA and ZIKV. The *SORD-like* construct was prepared and delivered using the same vector, cloning procedure, injection conditions, and empty-vector control as described for *CYP4G15*. Ovaries were collected at 2 and 4 days post-injection to validate overexpression and measure *CYP4G15* and *SORD-like* transcript levels by qPCR. For compensation assays, over*CYP4G15* + ds*SORD-like* + ZIKV and over*SORD-like* + ds*CYP4G15* + ZIKV groups were analyzed at day 4. Each biological replicate contained 30 ovaries (N = 3). ZIKV-positive ovaries identified by endpoint RT-PCR were further analyzed by qPCR to determine viral RNA copy numbers. This design enabled evaluation of both directional association and functional compensation between the two genes.

### 2.13 Statistical analysis

All experiments were performed with at least three independent biological replicates unless otherwise stated. Relative gene expression data are presented as mean ± standard error of the mean (SEM). Transcript abundance was normalized to *Aalrps7* and calculated using the 2^−ΔΔCt method (Livak and Schmittgen, 2001). Viral RNA copy numbers were analyzed either as raw values or after log10 transformation, depending on data distribution and figure presentation.

Statistical analyses were performed using SPSS 27.0. Normality was assessed using the Shapiro–Wilk test, and homogeneity of variance was evaluated when appropriate. For quantitative data meeting parametric assumptions, one-way analysis of variance (ANOVA) followed by Tukey’s multiple-comparison test was used. For comparisons involving unbalanced groups, Brown-Forsythe test was first performed to confirm homogeneity of variances. For proportion data, chi-square tests or Fisher’s exact tests were used as appropriate. A value of *p* < 0.05 was considered statistically significant.

## 3 Results

### 3.1 *CYP4G15* exhibits distinct spatiotemporal and infection-responsive expression patterns in *Aedes albopictus*

To explore the potential involvement of *CYP4G15* in ZIKV infection, we first characterized its expression patterns across tissues, the gonotrophic cycle, developmental stages, and in response to viral infection.

**Figure 1:**
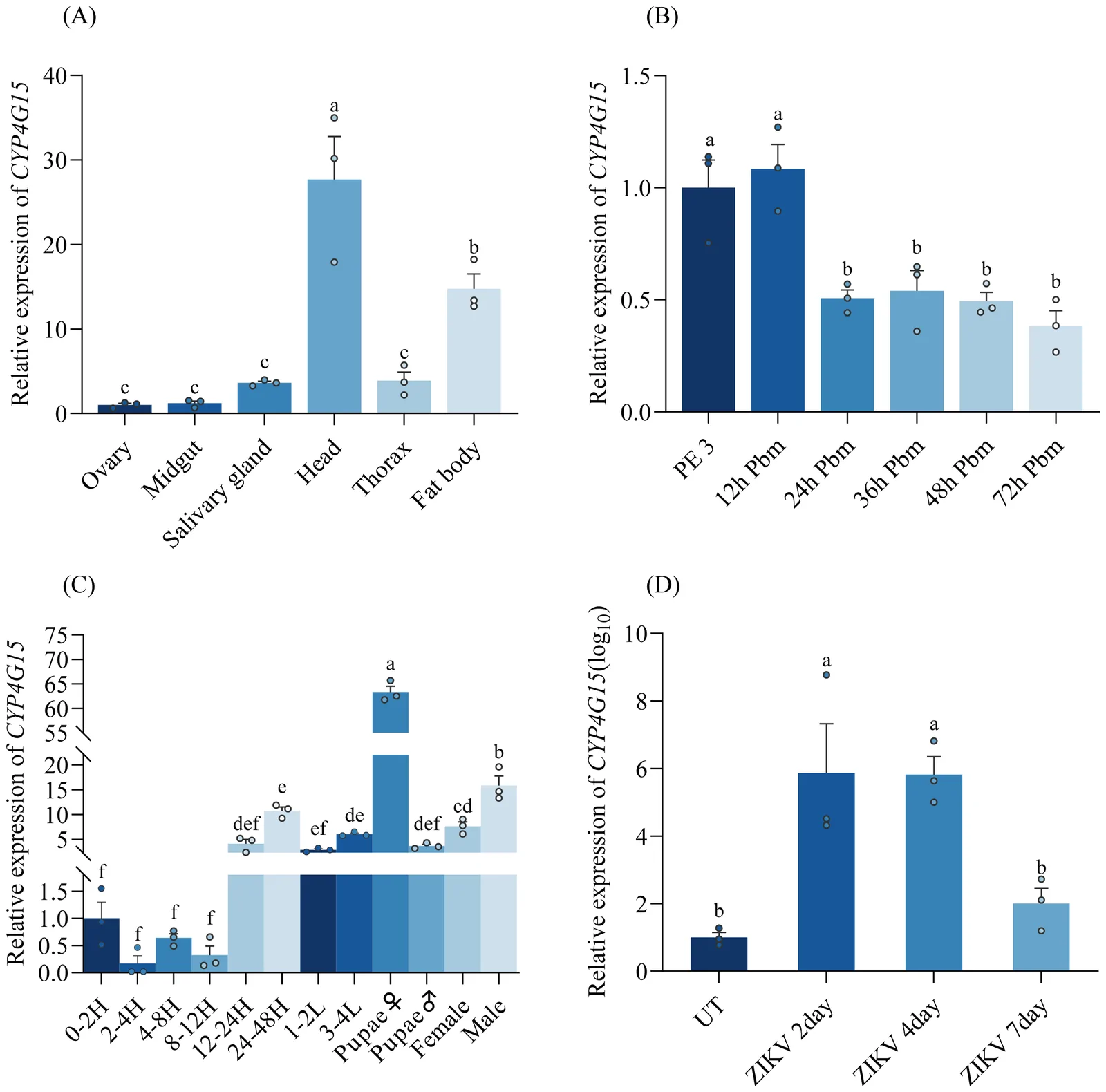
Tissue-, blood-feeding-, developmental-, and infection-responsive expression profiles of *CYP4G15* in *Aedes albopictus* **(A)** Tissue distribution of *CYP4G15* transcripts in non-blood-fed adult females, including ovary, midgut, salivary gland, head, thorax, and fat body. **(B)** Expression dynamics of *CYP4G15* before blood feeding (72 h post-emergence) and at 12, 24, 36, 48, and 72 h post-blood meal (PBM). **(C)** Developmental expression profile of *CYP4G15* across eggs, larvae, pupae, and adults. **(D)** Ovarian expression of *CYP4G15* at 2, 4, and 7 days after intrathoracic ZIKV injection. Transcript abundance was measured by RT-qPCR, normalized to *Aalrps7*, and calculated using the 2^−ΔΔCt method. Data are presented as mean ± SEM from three independent biological replicates. Statistical significance was determined by one-way ANOVA followed by Tukey’s multiple-comparison test. Different lowercase letters indicate significant differences among groups (*p* < 0.05); ns, not significant. Each dot in panel **(D)** represents a pool of 20 ovaries.

In adult females, *CYP4G15* showed a marked tissue-biased expression profile (**Figure 1A**). Transcript levels were highest in the head, followed by the fat body, whereas expression in the ovary, midgut, salivary gland, and thorax remained low. These findings indicate clear spatial specificity in *CYP4G15* expression.

During the blood-feeding cycle, *CYP4G15* expression remained high before blood feeding and at 12 h post-blood meal (PBM), but decreased significantly from 24 h onward (**Figure 1B**). Thus, *CYP4G15* appears to be preferentially associated with the pre-blood-feeding and early post-blood-feeding phases.

Developmental profiling further revealed strong stage-dependent regulation of *CYP4G15* (**Figure 1C**). Transcript abundance was minimal in early eggs, increased during late embryonic and larval stages, and peaked in female pupae. Expression in male pupae was substantially lower, whereas adult males showed higher levels than adult females. These results indicate tight developmental and sex-dependent control of *CYP4G15* expression.

We next examined ovarian expression of *CYP4G15* after ZIKV infection. Although basal expression of this gene in ovaries was low, transcript levels increased significantly at 2 and 4 days post-infection before declining at day 7 (**Figure 1D**). Collectively, these data demonstrate that *CYP4G15* exhibits dynamic spatiotemporal regulation and is inducible in ovarian tissue during the early phase of ZIKV infection, supporting its potential involvement in host responses associated with ovarian ZIKV infection.

### 3.2 Transcriptomic profiling after *CYP4G15* silencing identifies *SORD-like* as a candidate downstream effector

**Figure 2:**
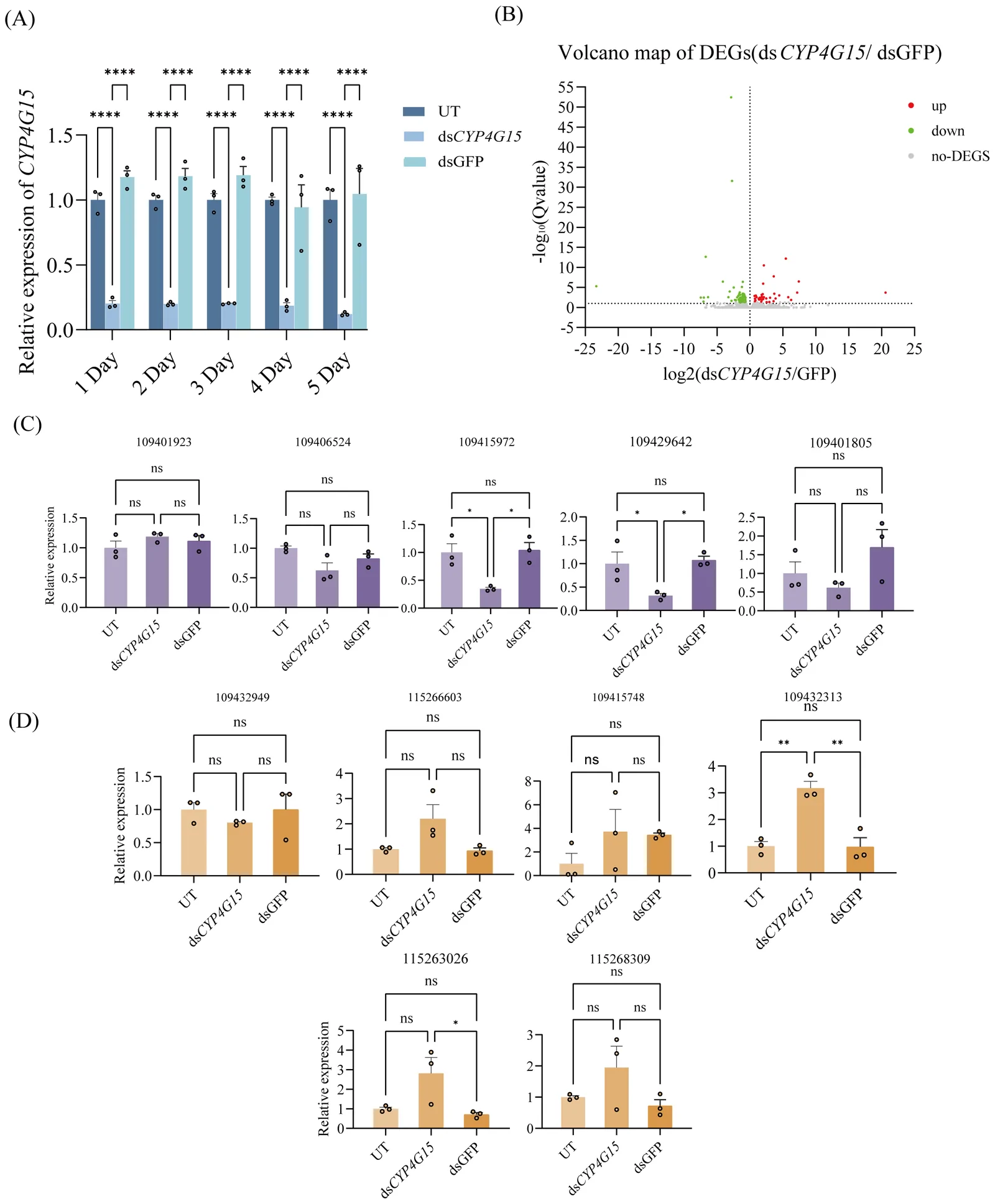
RNAi-mediated silencing of *CYP4G15* and identification of candidate downstream genes **(A)** Knockdown efficiency of *CYP4G15* at 1–5 days after intrathoracic injection of ds*CYP4G15*. dsGFP-injected and uninjected mosquitoes served as controls. Each data point represents RNA pooled from 8 adult females, with three independent biological replicates per group. **(B)** Volcano plot of differentially expressed genes identified by transcriptome sequencing after *CYP4G15* silencing. Red and green dots indicate significantly upregulated and downregulated genes, respectively, whereas gray dots indicate genes without significant differential expression. **(C)** RT-qPCR validation of five downregulated candidate genes: LOC109401923, LOC109406524, LOC109415972, LOC109429642, and LOC109431805. **(D)** RT-qPCR validation of six upregulated candidate genes: LOC109432949, LOC115266603, LOC109415748, LOC109432313, LOC115263026, and LOC115268309. Transcript abundance was measured by RT-qPCR, normalized to *Aalrps7*, and calculated using the 2^−ΔΔCt method. Data are presented as mean ± SEM from three independent biological replicates. Statistical significance was determined by one-way ANOVA followed by Tukey’s multiple-comparison test. *P < 0.05, **P < 0.01, and ****P < 0.0001; ns, not significant.

To investigate downstream genes regulated by *CYP4G15*, we first established an efficient RNA interference model in adult females. Three groups were included: ds*CYP4G15*-injected mosquitoes, dsGFP-injected mosquitoes as the negative control, and uninjected controls. Following intrathoracic injection, *CYP4G15* transcript levels were markedly reduced from day 1 and remained strongly suppressed through day 5 in the ds*CYP4G15* group compared with both control groups (**Figure 2A**). The dsGFP group showed expression levels comparable to those of uninjected mosquitoes, confirming sequence-specific silencing.

Day 3 post-injection was selected for transcriptome sequencing because knockdown efficiency was robust and inter-replicate variability was low at this time point. After quality filtering, clean reads were mapped to the *Aedes albopictus* reference genome. Differentially expressed genes were identified using the following criteria: |log2(fold change)| > 2, q < 0.05, and FPKM > 10. Genes showing excessive within-group variation or strong sequence homology to *CYP4G15* were excluded from further consideration, yielding 11 candidate genes, including five downregulated and six upregulated transcripts (**Figure 2B**).

RT-qPCR validation in independent samples confirmed significant downregulation of LOC109415972 (*SORD-like*) and LOC109429642, whereas LOC109401923, LOC109406524, and LOC109431805 did not show consistent changes (**Figure 2C**). Among the upregulated candidates, only LOC109432313 was reproducibly increased after *CYP4G15* silencing (**Figure 2D**). Thus, three genes showed concordant regulation between RNA-seq and RT-qPCR.

Among these validated candidates, LOC109415972, annotated as a sorbitol dehydrogenase-like gene (*SORD-like*), was prioritized for further study. It showed reproducible downregulation after *CYP4G15* silencing, had the highest transcript abundance (FPKM) among the three validated candidates, and was functionally linked by annotation to carbohydrate metabolism. By contrast, LOC109432313 has no clear functional annotation, whereas LOC109429642 encodes a retinaldehyde-binding protein and appeared less directly relevant to the present infection model. Taken together, these data identify *SORD-like* as a strong candidate downstream effector of *CYP4G15* in *Ae. albopictus*.

### 3.3 *SORD-like* exhibits ovary-associated, blood-feeding-, developmental-, and infection-responsive expression patterns in *Aedes albopictus*

After prioritizing *SORD-like* (LOC109415972) as a candidate downstream effector, we next characterized its expression profile across multiple biological contexts. Sequence annotation predicts that *SORD-like* encodes a sorbitol dehydrogenase-like protein, suggesting a potential role in carbohydrate metabolism. Given the close association between metabolic remodeling, mosquito reproduction, and arbovirus infection, we examined its expression across tissues, during the gonotrophic cycle, throughout development, and in response to ZIKV challenge.

**Figure 3:**
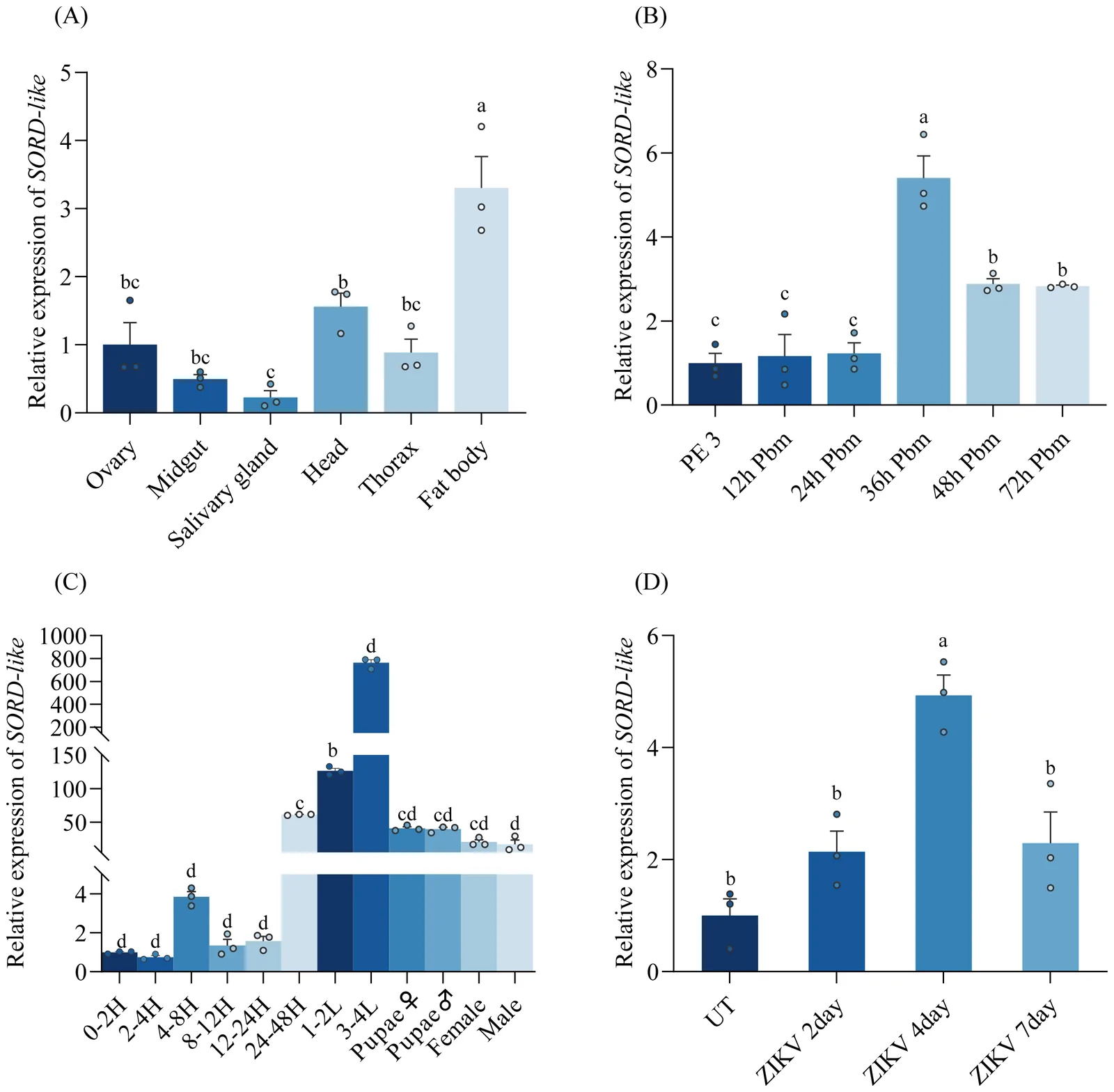
Tissue-, blood-feeding-, developmental-, and infection-responsive expression patterns of *SORD-like* in *Aedes albopictus* **(A)** Tissue distribution of *SORD-like* transcripts in non-blood-fed adult females, including ovary, midgut, salivary gland, head, thorax, and fat body. **(B)** Expression dynamics of *SORD-like* before blood feeding (72 h post-emergence) and at 12, 24, 36, 48, and 72 h post-blood meal (PBM). **(C)** Developmental expression profile of *SORD-like* across eggs, larvae, pupae, and adults. **(D)** Ovarian expression of *SORD-like* at 2, 4, and 7 days after intrathoracic ZIKV injection. Each dot represents a pool of 30 ovaries, with three independent biological replicates per time point. Transcript abundance was measured by RT-qPCR, normalized to *Aalrps7*, and calculated using the 2^−ΔΔCt method. Data are presented as mean ± SEM from three independent biological replicates. Statistical significance was determined by one-way ANOVA followed by Tukey’s multiple-comparison test. Different lowercase letters indicate significant differences among groups (*p* < 0.05); ns, not significant.

In adult females, *SORD-like* displayed a clear tissue-biased expression pattern (**Figure 3A**). Transcript abundance was highest in the fat body, followed by the head, with intermediate levels in the ovary, whereas expression in the midgut, salivary gland, and thorax remained relatively low. Notably, unlike *CYP4G15*, which showed very low basal expression in the ovary, *SORD-like* displayed moderate and readily detectable ovarian transcript levels, suggesting a more prominent association with ovarian physiology under non-infected conditions.

During the gonotrophic cycle, *SORD-like* expression remained relatively low before blood feeding and during the early post-blood-meal period, but increased sharply and reached a peak at 36 h post-blood meal (PBM) (**Figure 3B**). Expression then declined at 48 and 72 h PBM, although it remained significantly higher than pre-blood-feeding levels. This temporal pattern is consistent with a role for *SORD-like* during the metabolically active phase of post-blood-meal reproductive development.

Developmental profiling further revealed dynamic regulation of *SORD-like* throughout the mosquito life cycle (**Figure 3C**). Transcript abundance was low during embryogenesis, increased modestly during later egg stages, rose sharply during larval development, and reached its highest level in third- to fourth-instar larvae. Expression then decreased substantially during pupal and adult stages. These data indicate that *SORD-like* is most abundant during periods of high developmental and metabolic activity.

Because subsequent functional experiments focused on ovarian ZIKV infection, we further assessed whether *SORD-like* responded to viral challenge. Following intrathoracic ZIKV injection, ovarian *SORD-like* expression showed a modest but non-significant increase at 2 days post-infection, reached a significant peak at 4 days post-infection, and declined by day 7 (**Figure 3D**). This infection-responsive pattern, together with its moderate ovarian expression and strong induction after blood feeding, supports *SORD-like* as a physiologically relevant downstream effector associated with *CYP4G15* during ovarian ZIKV infection. Compared with the other validated transcriptomic candidates, the expression profile of *SORD-like* was most consistent with a role in post-blood-meal ovarian physiology and virus-associated host responses, further justifying its prioritization for functional studies.

### 3.4 *SORD-like* facilitates early ovarian infection and viral RNA accumulation of ZIKV in *Aedes albopictus*

To directly assess the functional role of *SORD-like* in ovarian ZIKV infection, adult females were intrathoracically co-injected with ds*SORD-like* and ZIKV. Three groups were compared: ds*SORD-like* + ZIKV, dsGFP + ZIKV as the negative control, and ZIKV only. Ovarian infection was evaluated at 4, 7, and 10 days post-infection by endpoint RT-PCR, followed by qPCR quantification of viral RNA in positive samples.

**Figure 4:**
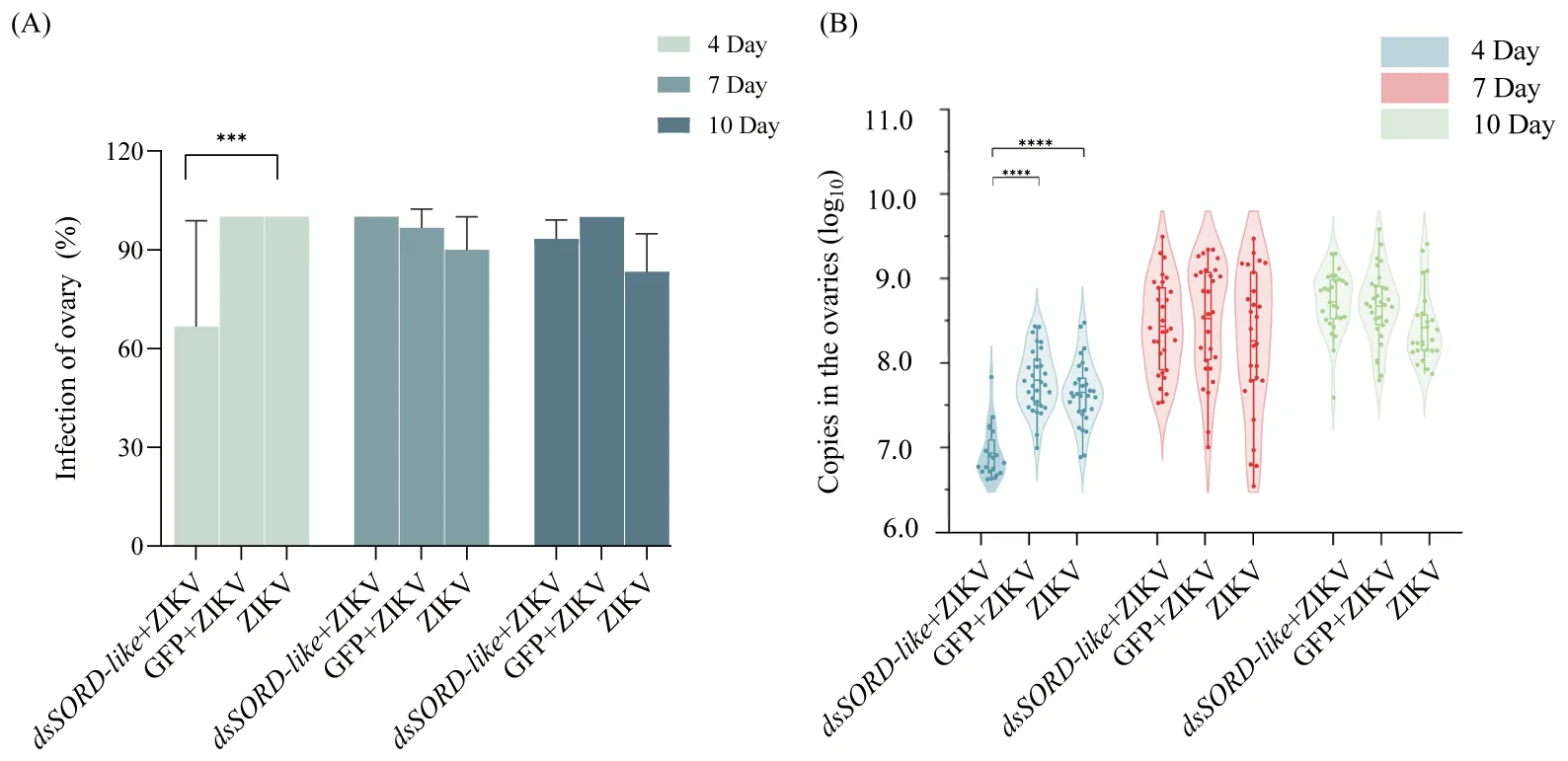
Silencing of *SORD-like* reduces early ovarian infection and viral RNA accumulation of ZIKV in *Aedes albopictus* **(A)** Ovarian infection rates at 4, 7, and 10 days after intrathoracic co-injection of ds*SORD-like* + ZIKV, dsGFP + ZIKV, or ZIKV only. Infection was determined by endpoint RT-PCR. For each group and time point, 30 individual ovaries were examined (10 per biological replicate, N = 3). **(B)** ZIKV RNA copy numbers in ovaries at 4, 7, and 10 days post-infection. Viral loads were quantified by qPCR using a plasmid-derived standard curve and are shown as log10-transformed values. Each dot represents one endpoint RT-PCR-positive ovary from an individual mosquito. The number of samples included in panel **(B)** corresponds to the positive counts reported for panel **(A)**. For each treatment group and time point, 30 individual ovaries were examined across three independent biological replicates, with 10 ovaries per replicate. The positive/total counts for the ds*SORD-like* + ZIKV, dsGFP + ZIKV, and ZIKV-only groups were 20/30, 30/30, and 30/30 at day 4; 30/30, 29/30, and 27/30 at day 7; and 28/30, 30/30, and 25/30 at day 10, respectively. Panel **(B)** includes only endpoint RT-PCR-positive ovaries and therefore represents viral RNA abundance conditional on ovarian positivity rather than overall ovarian viral burden. Statistical significance was determined by one-way ANOVA followed by Tukey’s multiple-comparison test. ***P < 0.001 and ****P < 0.0001; ns, not significant.

At 4 days post-infection, *SORD-like* knockdown significantly reduced the ovarian infection rate compared with both control groups (**Figure 4A**). No significant differences were observed at 7 or 10 days post-infection, indicating that the effect of *SORD-like* knockdown was most pronounced during the early phase of ovarian infection.

Consistent with the infection-rate data, among ZIKV-positive ovaries, viral RNA copy numbers in ovaries were significantly lower in the ds*SORD-like* group at day 4 post-infection (**Figure 4B**). This difference diminished at later time points, with viral RNA levels in the knockdown group becoming comparable to those in the controls. These results indicate that *SORD-like* is primarily required for efficient early establishment of ovarian infection. However, because RNAi-mediated silencing is transient, the diminished effect at later time points may also reflect the gradual loss of knockdown efficiency, and we cannot exclude the possibility that *SORD-like* may also contribute to later stages of infection if silencing were sustained.

The concordant reduction in both infection rate and viral RNA burden at the early stage suggests that *SORD-like* promotes rapid ZIKV expansion within reproductive tissues. Although silencing of this gene did not completely block ovarian infection, it delayed viral accumulation during a critical early time window. This phenotype is consistent with the idea that *SORD-like* facilitates efficient ovarian colonization by ZIKV and may therefore influence whether the virus reaches the threshold required for subsequent transmission to developing eggs.

Taken together, these findings identify *SORD-like* as an important host factor that facilitates early ovarian ZIKV infection in *Aedes albopictus*. Rather than being essential for ultimate viral persistence, *SORD-like* appears to modulate the kinetics of infection establishment, a feature likely to be important for downstream vertical transmission.

### 3.5 *SORD-like* knockdown reduces egg-associated ZIKV RNA detection in *Aedes albopictus*

Because ovarian infection is required for potential transovarial passage, we next tested whether knockdown of *SORD-like* affects ZIKV RNA detection in progeny egg pools. Mated adult females were intrathoracically co-injected with ds*SORD-like* and ZIKV, allowed to blood-feed, and then monitored individually for oviposition. Only egg batches derived from females with ZIKV-positive ovaries were included in the egg-pool analysis.

**Figure 5:**
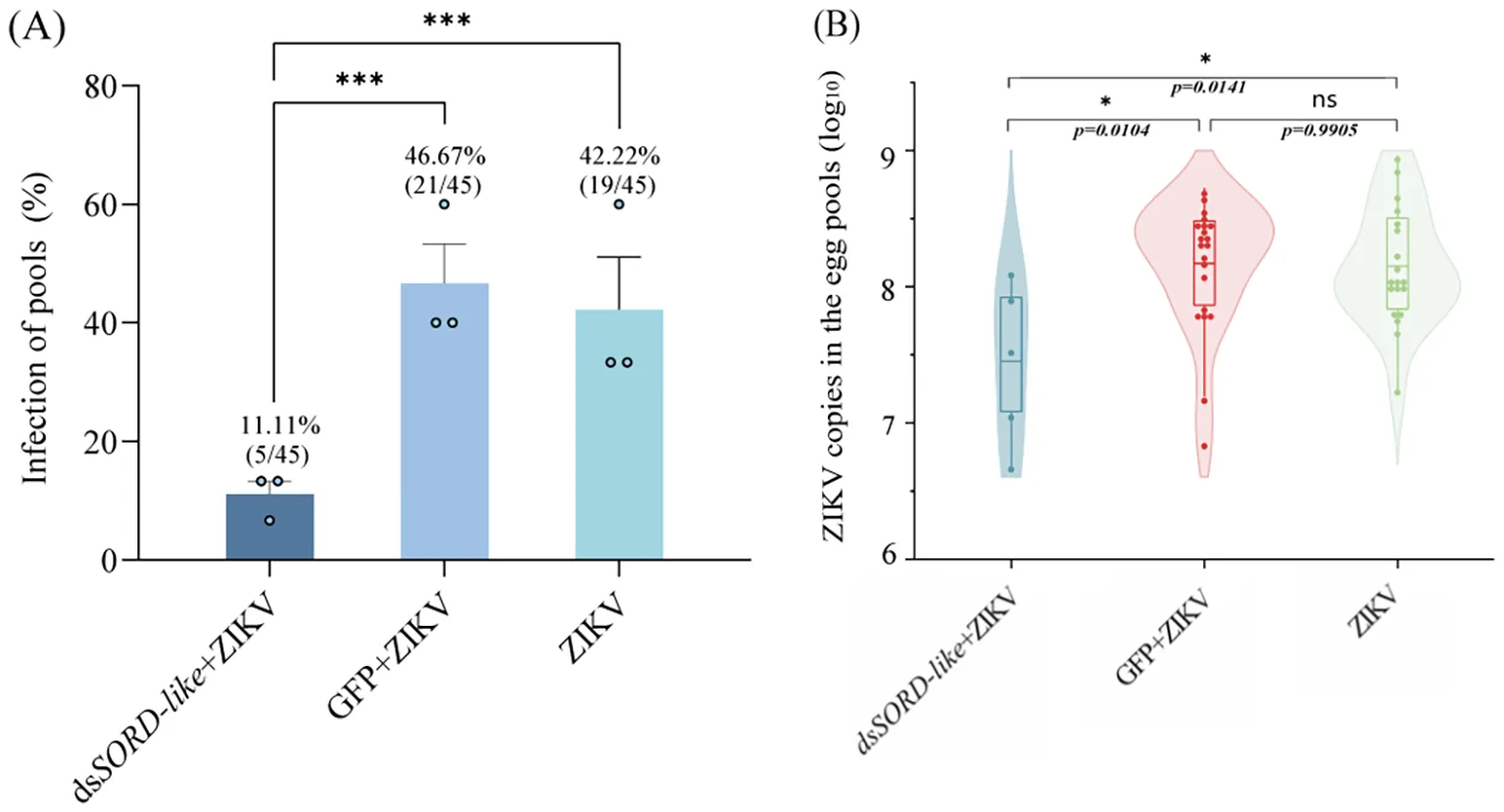
Effect of *SORD-like* knockdown on conditional egg-pool ZIKV RNA positivity and viral RNA abundance. **(A)** Conditional ZIKV RNA-positive egg-pool rate among egg batches derived from females with ZIKV-positive ovaries. Each treatment group included 45 egg pools across three independent biological replicates, with 15 pools per replicate. Each egg pool contained eggs from one mother and was treated as the observational unit. The positive/negative egg-pool counts were 19/26 for ZIKV only, 21/24 for dsGFP + ZIKV, and 5/40 for ds*SORD-like* + ZIKV. **(B)** Viral RNA copy numbers among ZIKV RNA-positive egg pools (ZIKV only, n = 19; dsGFP + ZIKV, n = 21; ds*SORD-like* + ZIKV, n = 5). Data represent viral RNA abundance conditional on egg-pool positivity. Viral RNA values were log10-transformed and analyzed by one-way ANOVA followed by Tukey’s multiple-comparison test. Homogeneity of variance was confirmed by Brown–Forsythe test (P = 0.7255). Exact adjusted P values, mean differences, and 95% confidence intervals are reported in the Results. *P < 0.05 and ***P < 0.001; ns, not significant.

*SORD-like* knockdown significantly reduced the conditional egg-pool positivity rate compared with both control groups (**Figure 5A**). Relative to the dsGFP + ZIKV group, the odds ratio was 0.143 (95% CI, 0.048–0.429; P = 0.0002), and relative to the ZIKV-only group, the odds ratio was 0.171 (95% CI, 0.057–0.515; P = 0.0008). No difference was detected between the dsGFP + ZIKV and ZIKV-only groups (OR = 1.197, 95% CI, 0.521–2.753; P = 0.6714). Among ZIKV RNA-positive egg pools, the overall one-way ANOVA was significant (F(2, 42) = 5.037, P = 0.0109). Tukey’s multiple-comparison test showed lower log10 viral RNA values in the ds*SORD-like* + ZIKV group than in the dsGFP + ZIKV group (mean difference = −0.7166, 95% CI, −1.285 to −0.1482; adjusted P = 0.0104) and the ZIKV-only group (mean difference = −0.6970, 95% CI, −1.271 to −0.1229; adjusted P = 0.0141). No difference was detected between the dsGFP + ZIKV and ZIKV-only groups (mean difference = 0.0196, 95% CI, −0.3421 to 0.3813; adjusted P = 0.9905). These results indicate that *SORD-like* knockdown reduces both egg-pool positivity and viral RNA levels within positive egg pools.

Although ovarian positivity rates remained high in all infected groups, the minimum egg infection rate was markedly lower in the ds*SORD-like* group (0.26%) than in the dsGFP + ZIKV group (1.00%) and the ZIKV-only group (1.14%) (**Table 1**). The number of ZIKV RNA-positive egg pools was also substantially reduced, despite broadly comparable maternal ovarian positivity rates.

**Table 1:** Maternal ovarian positivity and conditional egg-pool ZIKV RNA detection after *SORD-like* knockdown.

Parameter	ZIKV only	dsGFP + ZIKV	ds*SORD-like* + ZIKV
ZIKV-positive maternal ovaries	45/45	45/46	45/48
Ovarian positivity rate	100%	97.83%	93.75%
ZIKV RNA-positive egg pools	19/45	21/45	5/45
Conditional egg-pool positivity	42.22%	46.67%	11.11%
Total eggs examined	1, 665	2, 093	1, 956
Eggs represented in ZIKV RNA-positive pools	790	977	232
Minimum egg infection rate	1.14%	1.00%	0.26%
Eggs per pool, range (median)	1-71(38)	3-114(50)	1-95(45)

Only egg batches from females with ZIKV-positive ovaries were included; therefore, egg-pool positivity represents a conditional outcome rather than an overall vertical-transmission rate. Each egg pool contained eggs from one female. “Eggs represented in ZIKV RNA-positive pools” refers to the total number of eggs contained in positive pools and does not indicate individual egg positivity. Minimum egg infection rate was calculated as the number of positive egg pools divided by the total number of eggs represented in the tested pools × 100%. Eggs were surface-decontaminated with sodium hypochlorite before RNA extraction.

This phenotype is consistent with the early ovarian infection defect observed above. *SORD-like* knockdown reduced ovarian infection rate and viral RNA accumulation during the early phase of infection, although this effect diminished at later time points. These data suggest that timely establishment of ovarian infection may influence subsequent ZIKV RNA detection in progeny egg pools. In other words, successful transgenerational spread may depend not only on whether ZIKV reaches the ovary, but also on whether it achieves sufficient levels within the developmental window when oocytes are most susceptible.

Taken together, these findings indicate that *SORD-like* knockdown reduces ZIKV RNA detection in egg pools derived from ovarian-positive females. Rather than simply determining the final viral burden in already infected eggs, *SORD-like* may act as a factor influencing infection timing and transmission efficiency.

### 3.6 *CYP4G15* perturbation is associated with *SORD-like* transcript abundance and the ovarian ZIKV phenotype

To define the regulatory relationship between *CYP4G15* and *SORD-like*, we performed complementary loss- and gain-of-function experiments.

**Figure 6:**
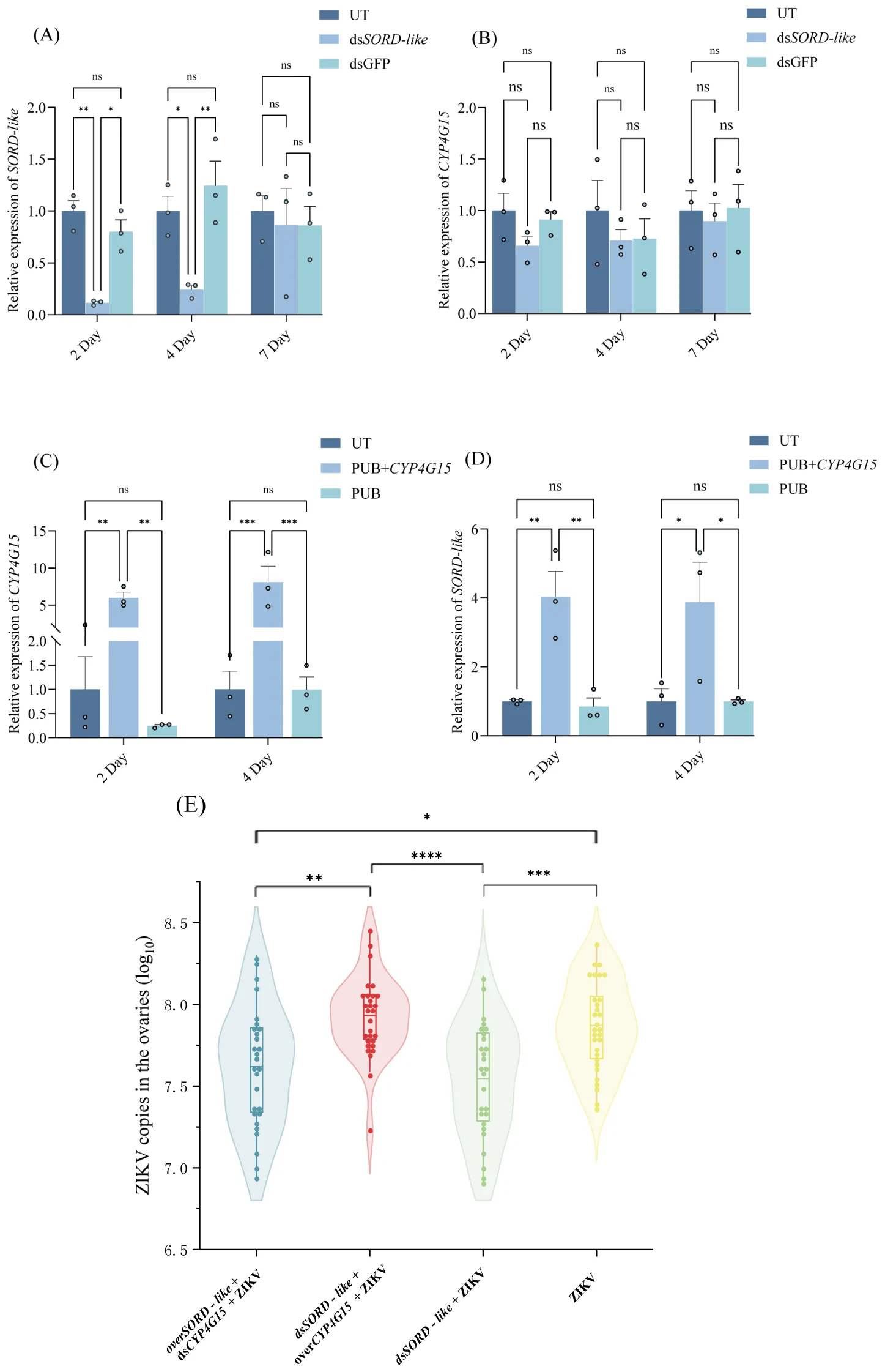
Association between *CYP4G15* perturbation, *SORD-like* transcript abundance, and the ovarian ZIKV phenotype in *Aedes albopictus* **(A)** Knockdown efficiency of *SORD-like* in ovaries at 2, 4, and 7 days after dsRNA injection. Each data point represents RNA pooled from 30 ovaries per biological replicate (N = 3). **(B)** Ovarian expression of *CYP4G15* after *SORD-like* knockdown. Data were obtained from the same RNA samples as in **(A)**. **(C)** Overexpression efficiency of *CYP4G15* at 2 and 4 days after intrathoracic injection of the *CYP4G15* expression plasmid. Each data point represents RNA pooled from 30 ovaries per biological replicate (N = 3). **(D)** Ovarian expression of *SORD-like* after *CYP4G15* overexpression. **(E)** Rescue-like assay showing ZIKV RNA copy numbers in individual ZIKV-positive ovaries at day 4 post-treatment. Groups: ZIKV only; ds*SORD-like* + ZIKV; ds*SORD-like* + over*CYP4G15* + ZIKV; over*SORD-like* + ds*CYP4G15* + ZIKV. Each group initially contained 30 individual ovaries from three independent biological replicates (N = 3). Ovaries were first screened by endpoint RT-PCR, and only ZIKV-positive ovaries were subjected to qPCR. The number of positive ovaries analyzed for each group was: ZIKV only, n = 30; ds*SORD-like* + ZIKV, n = 27; ds*SORD-like* + over*CYP4G15* + ZIKV, n = 29; over*SORD-like* + ds*CYP4G15* + ZIKV, n = 28. Each dot represents a single ZIKV-positive ovary. For panels **(A–D)**, transcript abundance was measured by RT-qPCR, normalized to *Aalrps7*, and calculated using the 2^−ΔΔCt method. For panel **(E)**, viral RNA copy numbers were quantified by qPCR and are shown as log10-transformed values. Data are presented as mean ± SEM from three independent biological replicates unless otherwise indicated. Statistical significance was determined by one-way ANOVA followed by Tukey’s multiple-comparison test. p < 0.01, *p* < 0.001; ns, not significant. *P < 0.05, **P < 0.01, ***P < 0.001, and ****P < 0.0001.

First, *SORD-like* was knocked down in adult females. Efficient silencing was confirmed at 2 and 4 days post-injection, whereas silencing efficiency declined by day 7 (**Figure 6A**). Despite the substantial reduction in *SORD-like* transcript levels at the early time points, ovarian *CYP4G15* expression remained unchanged relative to controls (**Figure 6B**). These results did not support an effect of *SORD-like* knockdown on *CYP4G15* transcript abundance.

We next tested whether elevated *CYP4G15* expression could influence SORD-like transcript abundance. Intrathoracic injection of the *CYP4G15* overexpression plasmid significantly increased *CYP4G15* transcript levels at 2 and 4 days post-injection compared with empty-vector and uninjected controls (**Figure 6C**). *CYP4G15* overexpression also significantly increased SORD-like transcript abundance at both time points compared with the corresponding controls (**Figure 6D**).

To assess the functional relevance of this relationship during infection, a rescue-like co-treatment assay was performed. Ovarian viral RNA accumulation was measured at 4 days post-infection. Knockdown of *SORD-like* alone (ds*SORD-like* + ZIKV) significantly reduced viral RNA levels compared with the ZIKV-only control. Co-injection of the *CYP4G15* overexpression plasmid together with ds*SORD-like* and ZIKV (ds*SORD-like* + over*CYP4G15* + ZIKV) restored viral RNA loads to levels comparable to the ZIKV-only control (**Figure 6E**). In contrast, overexpression of *SORD-like* in the *CYP4G15* knockdown background (over*SORD-like* + ds*CYP4G15* + ZIKV) failed to restore viral RNA accumulation.

Together with the reduction in *SORD-like* transcript abundance following *CYP4G15* knockdown, these findings support a directional functional association between *CYP4G15* and *SORD-like* during ovarian ZIKV infection. Because this study relied on RNAi-mediated knockdown rather than genetic knockout, residual target-gene expression cannot be excluded. Moreover, the rescue-like effect of *CYP4G15* overexpression after *SORD-like* knockdown may reflect residual *SORD-like* activity or additional *CYP4G15*-dependent pathways. Therefore, these data do not establish direct positive transcriptional regulation or a strict linear epistatic relationship. Accordingly, *SORD-lik*e is best regarded as a *CYP4G15*-associated factor within a broader host response during ovarian ZIKV infection.

## 4 Discussion

In this study, we identified a previously unrecognized host regulatory pathway involving cytochrome P450 4G15 (*CYP4G15*) and *SORD-like*, a functionally associated candidate, that promotes ovarian ZIKV infection and is associated with egg-associated viral detection in *Aedes albopictus*. *CYP4G15* displayed dynamic spatiotemporal and infection-responsive expression patterns, including transient induction in ovaries during the early phase of ZIKV infection. Transcriptomic profiling following *CYP4G15* silencing, together with RT-qPCR validation, identified *SORD-like* (LOC109415972) as a candidate downstream effector. Functional analyses further showed that *SORD-like* facilitates early ovarian infection, promotes viral RNA accumulation in reproductive tissues, and enhances transmission to progeny eggs. In addition, *CYP4G15* overexpression was associated with increased *SORD-like t*ranscript abundance and functionally compensated for the ovarian viral RNA phenotype caused by *SORD-like* knockdown. Together, these findings support a model in which a *CYP4G15*–*SORD-like*-associated pathway contributes to the timely establishment of ZIKV infection in reproductive tissues may thereby influence transgenerational viral transmission in *Ae. albopictus*. This framework extends current understanding of mosquito determinants of viral persistence beyond the better-studied midgut and systemic dissemination stages.

Vertical transmission is increasingly recognized as an important route by which arboviruses can persist in mosquito populations during inter-epidemic periods or under ecological conditions that reduce frequent mosquito–host contact (Lai et al., 2020; Janjoter et al., 2024). Although horizontal transmission remains the dominant route for ZIKV spread to vertebrate hosts, successful ovarian infection is a prerequisite for transovarial passage to the next generation (Lai et al., 2020). In *Ae. albopictus*, experimental work has shown that ZIKV can indeed be vertically transmitted, even if the overall transmission rate is relatively low (Lai et al., 2020). The relatively low vertical transmission rates observed in this study and in previous work raise an important ecological question: if transovarial transmission is typically inefficient, what is its epidemiological significance? We propose that the quantitative contribution of vertical transmission to viral maintenance should not be judged solely by the absolute infection rate of progeny eggs. Instead, the biological relevance of this route may be better understood in terms of its cumulative effect over multiple gonotrophic cycles and its role during periods when horizontal transmission is reduced. The *CYP4G15*–*SORD-like* pathway described here may serve as a host physiological axis that modulates the efficiency of this process by influencing the kinetics of ovarian infection. Even a moderate increase in transmission efficiency, when sustained across seasons or during inter-epidemic periods, could have a non-negligible impact on viral persistence in mosquito populations. Our study adds a mechanistic dimension to this framework by showing that the efficiency of vertical transmission is not determined simply by whether virus can eventually be detected in maternal ovaries, but also by whether ovarian infection is established rapidly enough to intersect with the reproductive window during which developing oocytes are susceptible. In this sense, the temporal dynamics of ovarian infection appear to be as important as the final ovarian viral burden.

One notable finding of this work is that *CYP4G15* behaves as a ZIKV-responsive host factor. Members of the *CYP4G* subfamily are unusual among insect P450s because they are classically associated with cuticular hydrocarbon biosynthesis, water balance, and chemical communication rather than with canonical detoxification (Qiu et al., 2012; Feyereisen, 2020). The best-established biochemical role of CYP4G enzymes is as oxidative decarbonylases involved in hydrocarbon formation (Qiu et al., 2012). Against this background, the transient induction of *CYP4G15* after ZIKV infection suggests that this gene is not merely a constitutively expressed physiological factor, but can also be recruited under infection conditions. This observation is particularly interesting in light of emerging evidence that P450-linked pathways may shape vector competence traits more broadly than previously appreciated (Huang et al., 2019; Wu et al., 2025). Our results therefore place *CYP4G15* at the interface between mosquito adaptive physiology and virus-associated host responses.

The expression profile of *CYP4G15* also provides clues to its broader biological context. In our data, *CYP4G15* showed pronounced spatial and temporal regulation, with highest expression in the head, relatively high expression in the fat body, dynamic changes after blood feeding, and strong developmental regulation. Such a pattern is consistent with the idea that *CYP4G15* is embedded in broader physiological programs linked to tissue specialization and life-stage transitions. Importantly, although CYP4G enzymes are not typically discussed as direct antiviral factors, P450 genes are central components of insecticide resistance-associated physiological adaptation in *Aedes* mosquitoes (Lien et al., 2019; Zulfa et al., 2022). In *Ae. aegypti*, *CYP4G15* itself has been reported as differentially expressed in an insecticide-resistant transcriptomic background, and more recent work has linked natural variation in cytochrome P450 regulation to dengue susceptibility (Lien et al., 2019; Merkling et al., 2025). Taken together with our results, these studies raise the possibility that certain P450-dependent adaptive pathways may influence both environmental stress tolerance and arbovirus infection phenotypes. We emphasize, however, that our study does not directly test insecticide resistance; rather, it suggests a plausible mechanistic bridge between these biological domains.

A second major advance of the present study is the identification of *SORD-like* as a downstream effector associated with *CYP4G15*. *SORD-like* is annotated as a sorbitol dehydrogenase-like gene, placing it within a putative metabolic context linked to sorbitol/fructose interconversion. Sorbitol dehydrogenases have long been recognized as important enzymes in carbohydrate metabolism, and insect homologs have been characterized in species such as *Drosophila* (Luque et al., 1998). Although the precise biochemical function of mosquito *SORD-like* remains to be experimentally validated, its expression profile in our study is biologically informative: it showed moderate ovarian expression, strong induction after blood feeding, and high expression during metabolically demanding developmental stages, especially larval stages. Blood feeding activates the vitellogenic phase of mosquito oogenesis and is accompanied by extensive metabolic remodeling in reproductive tissues and the fat body (Valzania et al., 2019; Lopez et al., 2024). Within this context, the marked post-blood-meal induction of *SORD-like* is consistent with a role in supporting the physiological transition required for ovarian maturation. This profile also made *SORD-like* a more compelling downstream candidate than the other validated differentially expressed genes.

Our results indicate that *SORD-like* is primarily associated for efficient early ovarian infection rather than for maintaining later-stage viral RNA accumulation. Silencing *SORD-like* significantly reduced ovarian infection rate and viral RNA burden at day 4 after infection, but this phenotype diminished at later time points. This temporal structure is one of the most important biological messages of the study. It suggests that *SORD-like* does not simply determine whether ZIKV can exist in ovarian tissue, but instead influences the speed with which the virus establishes infection during a critical early phase. We therefore favor a model in which *SORD-like* facilitates a host physiological state that permits rapid early viral expansion in reproductive tissues. Because our measurements are based mainly on ovarian positivity and viral RNA quantification, we deliberately avoid overinterpreting this as proof of a direct effect on intracellular replication per se. The phenotype could reflect changes in viral entry, early establishment, intracellular amplification, tissue permissiveness, or a combination of these processes. Nevertheless, the biological consequence is clear: loss of *SORD-like* delays the early accumulation of ZIKV in ovaries.

The temporal pattern of ovarian infection observed after *SORD-like* knockdown—with the effect significant only at day 4 post-infection and absent at days 7 and 10—is consistent with the reduced ZIKV RNA detection in progeny egg pools. Knockdown of *SORD-like* significantly reduced egg-pool positivity and markedly lowered the minimum egg infection rate; the viral RNA burden in positive eggs was also significantly reduced (**Figure 5B**). Thus, *SORD-like* appears to affect both the probability of successful viral passage into eggs and the subsequent viral RNA accumulation within eggs that have already become infected. This pattern suggests that *SORD-like* may contribute to ZIKV vertical transmission at more than one step. Our data are consistent with the idea that vertical transmission depends on whether virus reaches a sufficient level within a narrow developmental window before oviposition. If ovarian infection is delayed, even transiently, the virus may fail to enter enough oocytes in time, and the resulting egg positivity rate drops sharply. In this sense, *SORD-like* may act less as a determinant of ultimate viral persistence and more as a factor influencing infection timing and transmission efficiency. This interpretation is consistent with the transovarial transmission framework established for ZIKV in *Ae. Albopictus* (Lai et al., 2020; Janjoter et al., 2024).

Silencing *SORD-like* did not alter *CYP4G15* expression, whereas overexpression of *CYP4G15* increased *SORD-like* transcript abundance. Functional compensation experiments also supported this regulatory directionality: co-injection of the *CYP4G15* overexpression plasmid together with ds*SORD-like* and ZIKV restored ovarian viral RNA accumulation to levels comparable to the ZIKV-only control, whereas overexpression of *SORD-like* in the ds*CYP4G15* background failed to restore viral RNA accumulation. Together with the earlier observation that *CYP4G15* knockdown reduced *SORD-like* expression, these data support a *CYP4G15-SORD-like*-associated pathway during ovarian ZIKV infection, while allowing for residual *SORD-like* activity and additional *CYP4G15*-dependent pathways. Because the present study relied on RNAi-mediated knockdown rather than genetic knockout, the current data should be interpreted cautiously. They are strongest in supporting functional compensation under the tested conditions rather than establishing a strict linear epistatic relationship. Accordingly, *SORD-like* is best viewed as an important downstream effector within a broader host program controlled by *CYP4G15*, rather than the sole mediator of all *CYP4G15*-dependent phenotypes. This more conservative interpretation is also consistent with the increasingly complex view of vector competence, in which mosquito susceptibility reflects integrated physiological networks rather than single isolated genes (Huang et al., 2019; Wu et al., 2025).

The broader significance of this study lies in linking a *CYP4G*-associated host pathway to the persistence phase of arbovirus transmission biology. Recent work has suggested that mosquito adaptive traits—such as insecticide resistance, cuticular physiology, microbiome interactions, and metabolic state—can all influence vector competence in context-dependent ways (Moyes et al., 2017; Lien et al., 2019; Huang et al., 2019; Zulfa et al., 2022; Merkling et al., 2025; Wu et al., 2025). Our results extend this concept by suggesting that a host factor classically associated with adaptive physiology may also affect how efficiently ZIKV enters the vertical transmission route. This is particularly relevant for *Ae. albopictus*, one of the most invasive mosquito vectors worldwide and an increasingly important contributor to arbovirus emergence (Bonizzoni et al., 2013; Janjoter et al., 2024). If similar mechanisms operate under more natural oral infection conditions, such links could have practical implications: pathways traditionally studied in the context of vector ecology or insecticide adaptation may also shape long-term viral maintenance in mosquito populations.

Several limitations should be acknowledged. First, the infection model relied largely on intrathoracic inoculation, which is well suited for isolating systemic and ovarian stages of infection but bypasses the natural midgut barrier. Thus, the results may not fully recapitulate the complete oral infection process, particularly the midgut escape step, and extrapolation to natural transmission should be made with caution. Second, our viral measurements are based mainly on endpoint positivity and qPCR-derived RNA abundance, so the precise molecular step affected by *SORD-like*—viral entry, early establishment, intracellular amplification, or cell-to-cell spread—remains unresolved. Because eggs were tested as pooled samples and neither F1 progeny infection nor viral infectivity was assessed, the present findings demonstrate reduced ZIKV RNA detection in egg pools rather than confirmed reduction in transmission of infectious virus to the next generation. In addition, because the virus stock was quantified by RT-qPCR rather than an infectivity-based assay, the relationship between the administered viral RNA copy number and the infectious dose remains unknown. Third, the putative biochemical role of mosquito *SORD-like* in sorbitol/fructose metabolism was inferred from annotation rather than directly tested. Fourth, while the rescue-like assay supports a downstream role for *SORD-like*, it does not exclude the participation of additional *CYP4G15*-dependent effectors. Fifth, our time-course analysis revealed that the effect of *SORD-like* knockdown on ovarian infection was significant only at day 4 post-infection, with no detectable differences at days 7 and 10. While this temporal specificity informed the experimental design of subsequent vertical transmission and rescue-like assays, the longer-term effects of *SORD-like* on viral persistence in ovarian tissues and on progeny infectivity across multiple gonotrophic cycles were not examined in this study. Furthermore, because RNAi-mediated silencing is inherently transient, the diminished effect at later time points may partly reflect the gradual loss of knockdown efficiency, and we cannot exclude the possibility that *SORD-like* may also contribute to later stages of infection if silencing were sustained. These aspects remain to be addressed in future work. These limitations do not weaken the central conclusion of the study, but they define the most important next steps.

Future work should therefore focus on several directions. First, it will be important to validate the predicted metabolic activity of *SORD-like* directly and determine whether manipulating this gene alters sorbitol/fructose flux, redox balance, or other aspects of ovarian metabolic state. Second, infection assays using oral exposure and finer temporal sampling would help distinguish effects on early ovarian colonization from later maintenance. Additionally, longitudinal studies tracking vertical transmission across multiple gonotrophic cycles would help to assess the cumulative epidemiological impact of this pathway in natural settings. Third, tissue-specific approaches could clarify whether the key site of *SORD-like* action is the ovary itself, another reproductive compartment, or a more systemic metabolic tissue. Finally, given the growing evidence that *CYP4G15* and related physiological pathways can influence arbovirus susceptibility in mosquitoes (Merkling et al., 2025), it will be valuable to test whether the *CYP4G15*–*SORD-like* pathway also affects other arboviruses of *Ae. albopictus*, such as dengue or chikungunya viruses.

In summary, our study identifies a functional association between *CYP4G15*, *SORD-like* transcript abundance, and ovarian ZIKV infection in *Aedes albopictus*. *CYP4G15* perturbation was associated with directional changes in *SORD-like* transcript abundance, and *CYP4G15* overexpression functionally compensated for the ovarian viral RNA phenotype caused by *SORD-like* knockdown. These findings support a *CYP4G15–SORD-like*-associated pathway but do not establish direct transcriptional regulation or a strict linear epistatic relationship.

## Data Availability

The datasets presented in this study can be found in online repositories. The names of the repository/repositories and accession number(s) can be found below: https://www.ncbi.nlm.nih.gov/, PRJNA1478778.
