# Correction: The *CYP4G15*-*SORD*-like-associated pathway promotes ovarian ZIKV infection and egg-associated viral RNA detection in *Aedes albopictus*

**DOI:** 10.3389/fcimb.2026.1990923

**Published:** 2026-10-07

**Authors:** Mei Chen, Yajing Zhang, Wenwei Zhong, Mei Hong, Jinbao Gu, Zetian Lai

**Affiliations:** 1 Center of Laboratory Medicine, Shenzhen Nanshan People’s Hospital (Affiliated Sixth Hospital of Shenzhen University), Shenzhen, Guangdong, China; 2 School of Public Health, Southern Medical University, Guangzhou, Guangdong, China; 3 Medical Laboratory, The Third Affiliated Hospital of Shenzhen University, Shenzhen, Guangdong, China; 4 College of Laboratory Medicine, Dalian Medical University, Dalian, Liaoning, China; 5 Guangdong Provincial Key Laboratory of Infection Immunity & Inflammation, Department of Pathogen Biology, Shenzhen University Medical School, Shenzhen, Guangdong, China

**Keywords:** Aedes albopictus, CYP4G15, metabolic regulation, ovarian infection, SORD-like, vertical transmission, Zika virus

In the published article, affiliation 5 was erroneously omitted from the paper. The following affiliation was added for author Zetian Lai.

“5. Guangdong Provincial Key Laboratory of Infection Immunity & Inflammation, Department of Pathogen Biology, Shenzhen University Medical School, Shenzhen, Guangdong, China”

The original version of this article has been updated.

