# Supplementary material for: The CYP4G15-SORD-like-associated pathway promotes ovarian ZIKV infection and egg-associated viral RNA detection in Aedes albopictus
Source: Front Cell Infect Microbiol. 2026 Aug 27;16:1910501. doi: 10.3389/fcimb.2026.1910501 (PMC13558110; doi:10.3389/fcimb.2026.1910501)
Supplement: Supplementary file 1 [file DataSheet1.docx]

Supplementary Figure S1.

**Supplementary Figure S1. Overexpression efficiency of *SORD-like* in ovaries at 2 and 4 days after intrathoracic injection of the *SORD-like* expression plasmid.**

RNA was extracted from dissected ovarian tissues (30 ovaries per biological replicate, N = 3), and *SORD-like* transcript levels were measured by RT-qPCR, normalized to *Aalrps7*, and calculated using the 2⁻ΔΔCt method. Data are presented as mean ± SEM. Statistical significance was determined by one-way ANOVA followed by Tukey's multiple-comparison test. **p < 0.01; ***p < 0.001; ns, not significant. At 2 days post-injection, *SORD-like* expression in the over*SORD-like* group was significantly higher than both UT and PUB controls (p < 0.01). At 4 days post-injection, the difference was also significant (p < 0.001). No significant difference was observed between UT and PUB controls at either time point.
