# Supplementary material for: The CYP4G15-SORD-like-associated pathway promotes ovarian ZIKV infection and egg-associated viral RNA detection in Aedes albopictus
Source: Front Cell Infect Microbiol. 2026 Aug 27;16:1910501. doi: 10.3389/fcimb.2026.1910501 (PMC13558110; doi:10.3389/fcimb.2026.1910501)
Supplement: Supplementary file 2 [file Table1.docx]

**Supplementary Table 1. Primers used in this study**
This table summarizes all primers used for RT-qPCR, dsRNA synthesis, ZIKV detection and quantification, and plasmid construction. Primer sequences are shown in the 5′–3′ orientation. Repeated primers appearing in different experimental chapters were merged and listed once in the final table.

Table1-1. Primers used in this study

| Primer Name | Sequence (5′→3′) |
| --- | --- |
| *Aalrps7*-qF | ATGAACTCGGACCTGAAG |
| *Aalrps7*-qR | TTCTTGCTGTTGAACTCG |
| *CYP4G15*-qF | CCAACCGTCACTACTACGCA |
| *CYP4G15*-qR | CTTGAGCATGGCGTACTTGC |
| CYP4G15-F | GCGGATATCCCGGCAACATA |
| CYP4G15-R | CAGGAGTTTGACCTGTCGCT |
| *T7 CYP4G15*-F | GGATCCTAATACGACTCACTATAGGGCGGATATCCCGGCAACATA |
| T7 CYP4G15-R | GGATCCTAATACGACTCACTATAGGCAGGAGTTTGACCTGTCGCT |
| *GFP* -F | AATGGGCACAAATTTTCTGTCAGT |
| *GFP* -R | CCGGACTTGTATAGTTCATCCATGC |
| *T7-GFP*-F | GGATCCTAATACGACTCACTATAGGAATGGGCACAAATTTTCTGTCAGT |
| *T7-GFP*-R | GGATCCTAATACGACTCACTATAGGCCGGACTTGTATAGTTCATCCATGC |
| Down109406524-qF | GGGATTGAAACTAGCATACGCC |
| Down109406524-qR | GTTCCCAACGCCTTTTTCTGG |
| Down109401923-qF | GCTGTCAAATCGCTCTCGCT |
| Down109401923-qR | ATGGGCTTTGGGCATAAACATCTTG |
| Down109431805-qF | CAAAAGTCATTCGACCTGCGT |
| Down109431805-qR | GAGTGATTGCCACCCGTTTC |
| Down109415972-qF | GGGATTGGGTAACGAGGACG |
| Down109415972-qR | CCAACGCCGCTGGAAAATCAT |
| Down109429642-qF | CAGCAACAATCCAGAAAAATGACC |
| Down109429642-qR | TCGAAGTTCCTTCCGGGCTA |
| UP109432313-qF | AAAACCGAGAACCGATGCCT |
| UP109432313-qR | TCGAGTTAGAACGCCATCGTTA |
| UP109415748-qF | ACAAACACCACAACCCCAGT |
| UP109415748-qR | CGCGTGTGGGAAATAGACCT |
| UP109432949-qF | GGTACACAACTCCGACCACAA |
| UP109432949-qR | AGCTTGAGAGCCGAACGAAC |
| UP115263026-qF | GCTTTTTGACCGTAACGCCTA |
| UP115263026-qR | GCCTTGTATCACTCCACCCC |
| UP115266603-qF | CATCTCTAAACCAACGGCGG |
| UP115266603-qR | GCTAGCACTGTGACAATTACAAAA |
| UP115268309-qF | GACGCGATACAATCCAGTTTGA |
| UP115268309-qR | CAACCCACATGGTCAAGGGA |
| *SORD-like*-qF | GGGATTGGGTAACGAGGACG |
| *SORD-like*-qR | CCAACGCCGCTGGAAAATCAT |
| *SORD-like*-F | AGCACTGTACGACGCAGAAA |
| *SORD-like*-R | TGAATCCTCCGCACAAGGTC |
| T7*SORD-like*-F | GGATCCTAATACGACTCACTATAGGAGCACTGTACGACGCAGAAA |
| T7-*SORD-like*-R | GGATCCTAATACGACTCACTATAGGTGAATCCTCCGCACAAGGTC |
| *Aalrps7*-qF | ATGAACTCGGACCTGAAG |
| *Aalrps7*-qR | TTCTTGCTGTTGAACTCG |
| *GFP* -F | AATGGGCACAAATTTTCTGTCAGT |
| *GFP* -R | CCGGACTTGTATAGTTCATCCATGC |
| *T7-GFP*-F | GGATCCTAATACGACTCACTATAGGAATGGGCACAAATTTTCTGTCAGT |
| *T7-GFP*-R | GGATCCTAATACGACTCACTATAGGCGGACTTGTATAGTTCATCCATGC |
| *ZIKV NS1*-F | ACCCAAGTCTTTAGCTGGGC |
| *ZIKV NS1*-R | CTGGTTCTTTCCTGGGCCTT |
| *ZIKV*-qF | GGAGAAGAAGAGACGAGGCG |
| *ZIKV*-qR | GATATGGCCTCCCCAGCATC |
| *Aalrps7*-F | GGGACAAATCGGCCAGGCTATC |
| *Aalrps7*-R | TCGTGGACGCTTCTGCTTGTTG |
| *pLB-*F | CGACTCACTATAGGGAGAGCGGC |
| *pLB-*R | AAGAACATCGATTTTCCATGGCAG |
| M13-F | CGCCAGGGTTTTCCCAGTCACGAC |
| M13-R | GAGCGGATAACAATTTCACACAG |
| *CYP4G15 1701*-F | ATGTCCGCGACCGTTGCAC |
| *CYP4G15 1701*-R | TCAGGCTTTCGCCGCTTTTG |
| *CYP4G15 1745*-F | TGCTCAACAGAGATTTCAACGCATGTCCGCGACCGTTGCACCGG |
| *CYP 4G15 1745*-R | ACATTCTTGGAAGACCTCATGCTCAGGCTTTCGCCGCTTTTGGTTT |
