# Supplementary material for: The CYP4G15-SORD-like-associated pathway promotes ovarian ZIKV infection and egg-associated viral RNA detection in Aedes albopictus
Source: Front Cell Infect Microbiol. 2026 Aug 27;16:1910501. doi: 10.3389/fcimb.2026.1910501 (PMC13558110; doi:10.3389/fcimb.2026.1910501)
Supplement: Supplementary file 3 [file Table2.docx]

**Supplementary Table S2. Key reagents and resources.**

Table2-1.Key reagents

| Name of reagents | Company Affiliation |
| --- | --- |
| AG RNAex Pro Reagent(Cat.No AG21101) | Accurate Biology |
| Chloroform / Isopropanol / Anhydrous Ethanol | Guangdong Guanghua Sci-Tech Co., Ltd. |
| RNAse-free water(Cat.No BL510B) | biosharp |
| DreamTaq^TM^ Hot Start Green PCR Master Mix | Thermo Fisher Scientific |
| TransScript® One-Step gDNA Removal and cDNA Synthesis SuperMix(Cat.No：AT311) | TransGen Biotech Co., Ltd. |
| PerfectStart® Green qPCR SuperMix(+Universal Passive Reference Dye)(Cat.No：AQ602) | TransGen Biotech Co., Ltd. |
| T7 RiboMAX^TM^ Express RNAi system(Cat.No P1700) | Promega |
| SteadyPure PCR Purification Kit(Cat.No AG21003) | Accurate Biology |
| Fetal Bovine Serum (Prime)(Cat.No FSP500) | ExCell Bio |
| RPMI Medium 1640(Cat.No C11875500BT) | Gibco |
| Sterile Defibrinated Sheep Blood (Cat.No SBJ-ST-S001) | Senbeijia |
| Penicillin-Streptomycin （10,000U/mL）(Cat.No 15140122) | Gibco |
| Trypsin-EDTA（0.25%）(Cat.No 25200072) | Gibco |
| pLV3-U6-MCS-shRNA-EF1a-CopGFP-Puro | MiaoLingBio |
| EasyPure® EndoFree Pro Plasmid MaxiPrep Kit(Cat.No：EM122) | TransGen Biotech Co., Ltd |
| Thermo Scientific FastDigest XhoI(Cat.NoFD0694) | Thermo Fisher Scientific |
| FastPure® Gel DNA Extraction Mini Kit(Cat.No DC301) | Vazyme |
| pEASY®-Basic Seamless Cloning and Assembly Kit (Cat.No：CU201) | TransGen Biotech Co., Ltd |
| Takara PrimeSTAR® Max DNA Polymerase（Cat.No R045Q) | Takara |
| pLB Zero Background Rapid Cloning Kit (Cat.No VT205) | TIANGEN Biotech (Beijing) Co., Ltd. |
| rTaq DNA Polymerase ((Mg²⁺ & dNTP included)(Cat.No TAP-211) | Toyobo |
| TOP10 Competent Cells (Cat.No CB104) | TIANGEN Biotech (Beijing) Co., Ltd. |
| SteadyPure DNA Gel Extraction Kit(Cat.No AG21006) | Accurate Biology |
| SteadyPure PCR Purification Kit (Cat.No AG21003) | Accurate Biology |
| AG RNAex Pro Reagent(Cat.No AG21101) | Accurate Biology |

Table2-2. Equipment used in the experiment

| Name of Instrument and Equipment | Company Affiliation |
| --- | --- |
| Applied Biosystems QuantStudio 6 Flex | Thermo Fisher Scientific (USA) |
| Real-Time PCR System |  |
| Clean Bench (Model: SW-CJ-IF) | Sujing Antai Air Technology Co.,Ltd. |
| Biological Safety Cabinet (Model: SG403TXCE) | BARKER Inc., USA |
| Artificial Climate Incubator (Model: HP400GS) | Ruihua Instrument Equipment Co., Ltd. (Wuhan, China) |
| Programmable Horizontal Microelectrode Puller (Model: P-97) | SUTTER INSTRUMENT (USA) |
| Adjustable Vortex Mixer (Model: MS-S) | Scilogex LLC, USA |
| -80℃ Ultra-low Temperature Freezer | Haier Group (Qingdao, China) |
| -10℃~-25℃ Medical Low-temperature Storage Freezer | Haier Group (Qingdao, China) |
| Benchtop High-speed Refrigerated Centrifuge (Cat.No.5154R) | Eppendorf AG (Germany) |
| High-pressure Steam Autoclave | HIRAYAMA Manufacturing Corporation (Japan) |
| Electrophoresis Apparatus (Model: DYY-6D) | Beijing Liuyi Instrument Factory (Beijing, China) |
| Gel Doc 2000 Gel Documentation System | BIO-RAD Laboratories (USA) |
| Tissue Homogenizer | Tiangen Biotech (Beijing) Co., Ltd. (Beijing, China) |
| Microwave Oven | Galanz Group (Foshan, China) |
| Ice Maker | Panasonic Corporation, Japan |
| Hemitek Blood Feeding Unit | Discovery Workshops,Lancashire,UK |
| Analytical Electronic Balance (Model: FA1104) | Shanghai Balance Instrument Factory (Shanghai, China) |
